# SETD1A-SOX2 axis is involved in tamoxifen resistance in estrogen receptor α-positive breast cancer cells

**DOI:** 10.7150/thno.72599

**Published:** 2022-07-18

**Authors:** Ming Li Jin, Liu Yang, Kwang Won Jeong

**Affiliations:** 1 Gachon Research Institute of Pharmaceutical Sciences, College of Pharmacy, Gachon University, 191 Hambakmoero, Yeonsu-gu, Incheon, 21936, Republic of Korea; 2 Collaborative Innovation Center for Chinese Medicine and Respiratory Diseases Co-Constructed by Henan Province & Education Ministry of P.R. China, Henan University of Chinese Medicine, Zhengzhou, 450046, China

**Keywords:** breast cancer, tamoxifen resistance, SETD1A, SOX2, cancer stem cell

## Abstract

**Rationale**: Approximately 30-40% of estrogen receptor (ER)-positive breast cancer (BC) cases recur after tamoxifen therapy. Thus, additional studies on the mechanisms underlying tamoxifen resistance and more specific prognostic biomarkers are required. In this study, we investigated the role of the SET domain containing 1A (SETD1A), a histone H3-lysine 4 (H3K4) methyltransferase, in the development of tamoxifen resistance in BC.

**Methods**: The relationship between tamoxifen resistance and SETD1A protein level was investigated using resistant cell lines derived from the parent BC cells. Biochemical and molecular assays, such as RNA-sequencing, reverse transcription-quantitative polymerase chain reaction, chromatin-immunoprecipitation, and protein-binding assays, were used to identify the SETD1A target gene in tamoxifen-resistant BC cells. Additionally, the role of SETD1A in cancer stem cells (CSCs) was investigated using CSCs isolated from tamoxifen-resistant BC cells. Comprehensive transcriptome analysis and immunofluorescence staining using clinical datasets and tissue microarray were performed to determine the correlation between the expression of the SETD1A-SRY-box transcription factor 2 (SOX2) pair and recurrence in tamoxifen-treated patients with BC.

**Results**: SETD1A was expressed at higher levels in tamoxifen-resistant BC cells than in primary BC cells. Notably, SETD1A-depleted tamoxifen-resistant MCF-7 cells showed restored sensitivity to tamoxifen, whereas SETD1A overexpression in MCF-7 cells resulted in decreased sensitivity. SETD1A is recruited to the *SOX2* gene via its interaction with SOX2, thereby enhancing the expression of *SOX2* genes in tamoxifen-resistant BC cells. The growth of tamoxifen-resistant cells and CSCs was effectively suppressed by SETD1A knockdown. In addition, high levels of SETD1A and SOX2 were significantly correlated with a low survival rate in patients with ER-positive tamoxifen-resistant BC.

**Conclusion**: Our findings provide the first evidence of the critical role of the SETD1A-SOX2 axis in tamoxifen-resistant BC cells, implying that SETD1A may serve as a molecular target and prognostic indicator of a therapeutic response in patients with tamoxifen-resistant BC.

## Introduction

Tamoxifen is a representative selective estrogen receptor modulator (SERM) that inhibits the proliferation of breast cancer (BC) cells by competitive antagonism of estrogen receptor (ER)-mediated transcription. After approval for use in patients with advanced BC, tamoxifen has been widely used as the gold standard for the treatment of BC at all stages 1. However, approximately 30-40% of patients treated with tamoxifen for five years develop acquired resistance, which remains a major challenge in BC therapy. Early studies have shown that alterations in ER signaling by downregulation or mutation of ERα are potential mechanisms for acquired resistance 2-4. Indeed, approximately 1% of all primary tumors carry ERα mutations 5, and approximately 15-20% of SERM-resistant BC arise from the loss of ER expression 6. Several phenotypic changes besides ER have been reported in tamoxifen resistant (TamR) BC cells 6, 7. Enhanced expression levels of epidermal growth factor receptor (EGFR), erb-b2 receptor tyrosine kinase 2, and insulin like growth factor 1 receptor as well as activation of the downstream signaling pathway components, extracellular signal-regulated kinase and phosphatidylinositol 3-kinase, are major changes that accompany tamoxifen resistance 8-10. Moreover, reduced expression of p21 or p27 and overexpression of cyclin D1, cyclin E1, and MYC reduce estrogen sensitivity *in vitro* 11-14.

Recent studies have reported the association between tamoxifen resistance and enhanced expression levels of stem cell factors 15-17 For instance, SRY-box transcription factor 2 (SOX2) is aberrantly expressed in lung, brain, ovary, bone, colon, skin, and BC cells 18-24. BC cells overexpressing SOX2 are highly proliferative, invasive, and tumorigenic 15, 25 and showed frequent resistance to chemotherapeutic drugs in clinical settings, resulting in poor prognosis in patients with BC 26. However, the exact mechanism underlying the role of stem cell factors in endocrine resistance to ER-targeted SERM, such as tamoxifen, remains unclear.

Epigenetic regulators are considered one of the major drivers of anticancer drug resistance 7, 27. For example, DNA hypermethylation of *MLH1, Gas1, STA, C8orf4, LAMB3, TUBB, G0S2*, and *MCAM* gene promoter regions has been shown to be involved in acquired resistance to cytotoxic chemotherapeutic drugs in several tumor cell line models 28-32. In addition, inhibition of the histone H3K27 methyltransferase EZH2 in various cancers reduces the drug-resistant stem cell population 33-35. Bromodomain inhibitor also increased the apoptosis of T-ALL cells resistant to γ-secretase inhibitor treatment 36. Lung cancer cells that are resistant to tyrosine kinase inhibitors can be killed by histone deacetylase (HDAC) inhibitors 37. SETD1A, a histone H3K4-specific methyltransferase, promotes the proliferation of metastatic castration-resistant prostate cancer by regulating the expression of forkhead box M1 and octamer-binding transcription factor 4 (OCT-4) 38. In addition, our previous study demonstrated the involvement of SETD1A in the expression of genes characteristically expressed in TamR cancer cells (for example, EGFR) as well as ERα-mediated target gene expression 39.

Here, we investigated the mechanism underlying the involvement of SETD1A in the acquisition of tamoxifen resistance in ERα-positive BC cells. First, we measured the changes in SETD1A protein levels following tamoxifen resistance progression. Additionally, we investigated the role of SETD1A overexpression in the development of tamoxifen resistance. Further, we revealed that SETD1A regulates specific transcription factors required for maintaining the pluripotency of cancer stem cells in TamR BC cells. Our findings suggest that SETD1A plays a crucial role in the acquisition of tamoxifen resistance in ER-positive BC cells.

## Materials and Methods

### Cell culture, plasmids, and siRNAs

MCF-7 cells were cultured in high-glucose Dulbecco's modified Eagle's medium (DMEM) supplemented with 10% fetal bovine serum (FBS). T47D cells were grown in Roswell Park Memorial Institute-1640 medium supplemented with 10% FBS. TamR cells were produced by 12-month treatment of MCF-7 and T47D cells with 4-hydroxytamoxifen (Tamoxifen, 5 μM). All cells were incubated at 37 °C with 5% CO_2_ in a humidified incubator. MCF-7 and T47D cells were obtained from the Korean Cell Line Bank (KCLB, Seoul, Korea). Full-length SOX2 expression plasmid was purchased from Addgene (Watertown, MA, USA). pcDNA3-flag-SETD1A was kindly provided by Professor David Skalnik (Purdue University, IN, USA). The sequences of siRNAs and shRNAs are listed in Table S1.

### RNA-sequencing analysis

The total RNA purified from each group (n = 2) was processed to prepare an mRNA-seq library using the QuantSeq3 mRNA-seq Library Prep kit (Lexogen, Vienna, Austria). Each library was sequenced using an Illumina NextSeq500 instrument (Illumina, San Diego CA, USA). The original image data were converted into sequence data and stored in FASTQ format. Genes showing an absolute fold change (FC) of at least 1.5 between groups and *P* < 0.05 were considered to be differentially expressed. Gene set enrichment was measured via X2K analysis 40.

### Analysis of nascent mRNA

Newly synthesized RNA was labeled and isolated using a Click-iT Nascent RNA Capture Kit (Invitrogen, Carlsbad, CA, USA). Nascent RNAs were labeled with 0.2 mM 5-ethynyl uridine (EU) for 16 h and harvested. RNA was isolated from the cells using TRIzol reagent (Invitrogen, Carlsbad, CA, USA). A total of 1 μg of RNA was used for the click reaction, and 12 μL of Dynabeads (Invitrogen, Carlsbad, CA, USA) was used for 1 μg of biotinylated EU-RNA. Reverse transcription was performed using an iScript cDNA Synthesis Kit (Bio-Rad Laboratories, Hercules, CA, USA). Quantitative polymerase chain reaction (qPCR) was performed using using LightCycler 480 II instrument (Roche, Indianapolis, IN, USA) with the primers listed in Table S2.

### Tissue microarray and immunofluorescence staining

A human breast cancer tissue microarray (BRM961a) comprising normal breast tissue (n = 12) and invasive ductal carcinoma tissues (n = 47) was obtained from US Biomax Inc. (Rockville, MD, USA). Microarray slides were deparaffinized and incubated with anti-SETD1A (Bethyl Laboratories, Montgomery, AL, USA) and anti-SOX2 (Thermo Fisher Scientific, Waltham, MA, USA) primary antibodies. Anti-rabbit DyLight488 and anti-mouse DyLight594 (Vector Laboratories, Burlingame, CA, USA) antibodies were used as secondary antibodies (Table S3). The stained area was observed using a confocal microscope (Nikon, Tokyo, Japan) and the average integrated optical density (IOD) of SETD1A and SOX2 was measured at five randomly selected sites for each tissue sample using the ImageJ 1.8.0 software.

### Protein-protein interaction assay

For *in vitro* binding assay, His-SETD1A (SET domain) was purified using Ni-NTA agarose beads. Glutathione S-transferase (GST)-fused SOX2 protein (a.a. 1-317, 1-204, 1-120, and 1-40) and SET domains of SETD1A (Win-SET, a.a. 1450-1707 and SET, a.a. 1538-1707) were expressed in BL21-DE3 and purified using glutathione-agarose beads. Full-length SETD1A was synthesized using the TNT T7 Quick kit (Promega, Madison, WI, USA). For the co-immunoprecipitation assay, cell extracts were prepared using a lysis buffer (20 mM Tris-Cl [pH 8.0], 150 mM NaCl, 1% NP-40, and 2 mM EDTA) 41. The SETD1A antibody was immobilized on Protein A/G agarose beads (Santa Cruz Biotechnology, Dallas, TX, USA) and incubated overnight with cell extracts at 4 °C. The beads were washed with lysis buffer and analyzed via western blotting.

### Mammosphere formation assay

MCF-7 or TamR cells (200 cells/well) were suspended in mammosphere medium (20 ng/mL epidermal growth factor, 10 ng/mL basic fibroblast growth factor, 5 μg/mL insulin, 0.4% bovine serum albumin, B27 supplement, and 500 mL DMEM) in a 96-well ultra-low attachment plate (Corning Inc., Corning, NY, USA). The cells were placed in an incubator at 37 °C and 5% CO_2_ for seven days. Mammosphere number was counted under a microscope using 4× and 10× magnification. The percentage of mammospheres formed was calculated by dividing the number of mammospheres formed by the number of single cells plated.

### Data collection and analysis of the transcriptome datasets

The ROC Plotter platform (http://rocplt.org) 42 was used to evaluate the prognostic significance of SETD1A levels in tamoxifen-treated patients with BC. The data used to compare SETD1A and SOX2 mRNA levels in tamoxifen-treated patients with BC were obtained from microarray datasets (GSE9893). The R2 Genomics Analysis and Visualization platform (http://r2.amc.nl) was used to determine the correlation between SETD1A and SOX2 expression levels in BC datasets (GSE42568, GSE124648).

### Statistical analysis

Statistical analyses of the data from cell viability, migration, qPCR, protein correlation analysis, and tumor growth assays in a mouse xenograft model were conducted using GraphPad Prism software v8.0.2 (La Jolla, CA, USA). Statistical significance was determined using an unpaired Student's t-test. Survival analysis was performed using BreastMark 43.

## Results

### SETD1A is overexpressed in tamoxifen-resistant BC cells

**Figure 1:**
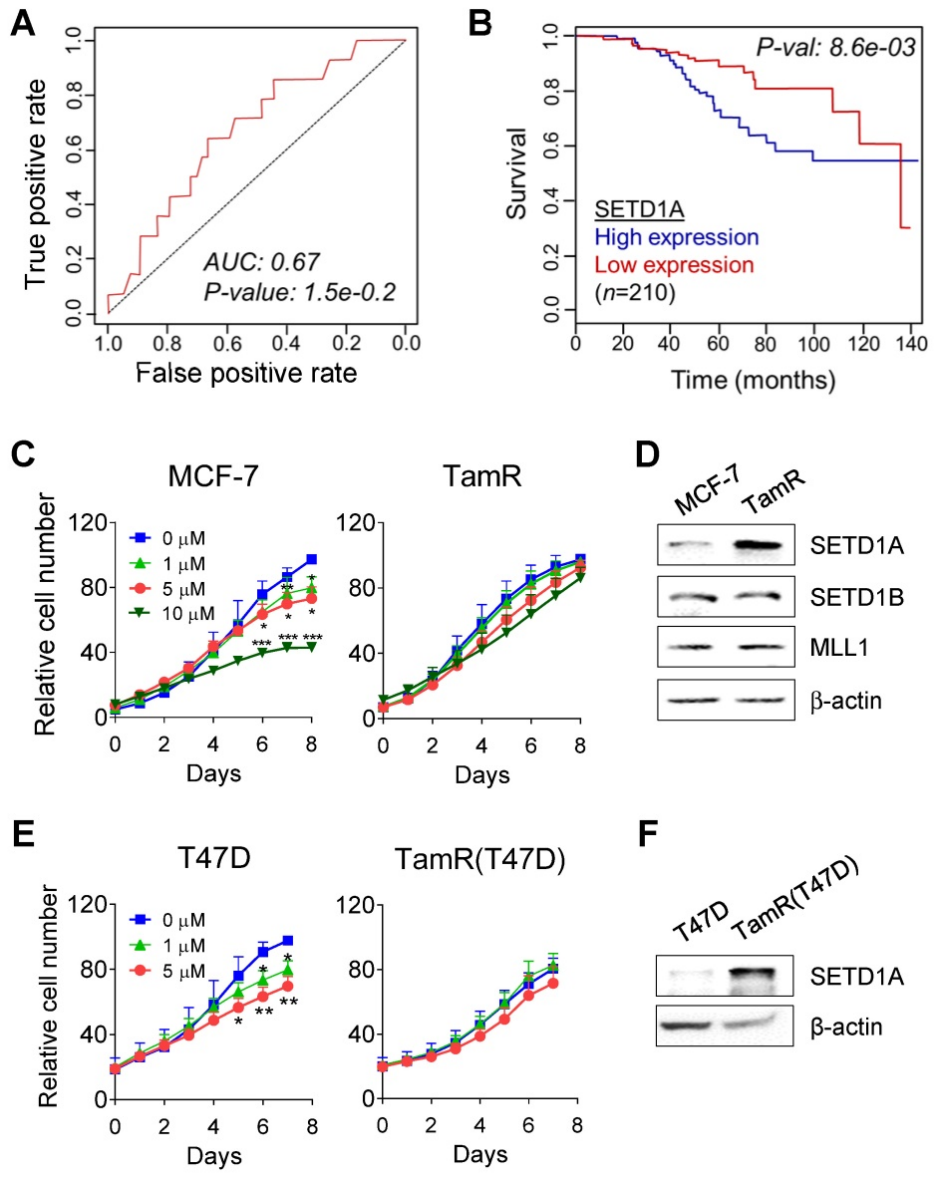
** SETD1A is overexpressed in tamoxifen-resistant breast cancer cells. (A)** Results of SETD1A expression analysis that predict the poor outcome in patients with breast cancer treated with tamoxifen. Area under the curve (AUC), *P*-value, false-positive rate (FPR), and true-positive rate (TPR) were calculated for SETD1A gene signature. **(B)** Kaplan-Meier survival curves indicating the relationship between SETD1A mRNA expression levels and survival rate were generated using *Breast Mark* filtered cancer datasets (n = 210). **(C, E)** Parent breast cancer (MCF-7 and T47D) and their tamoxifen-resistant (TamR and TamR(T47D)) cells were treated with vehicle or tamoxifen. Cell proliferation was assessed for eight days after treatment using a live cell imaging system. Data are expressed as the mean ± S.D. (n = 3). *P < 0.05; **P < 0.01; ***P < 0.001. **(D, F)** SETD1A expression levels in breast cancer and TamR cells were measured via western immunoblotting.

Our previous study confirmed that SETD1A mediates the survival of TamR cells 39. Therefore, we performed an online receiver-operating characteristic (ROC) analysis 42 to detect whether the expression of SETD1A affected the prognosis of patients with BC treated with tamoxifen. During the first five years after tamoxifen therapy in ER-positive, HER2-negative, and nodal-positive luminal B patients with BC, high expression of SETD1A was associated with a high rate of relapse (AUC 0.67, *P* = 1.5E-02; Figure 1A). When the analysis was performed using a cohort of ER-positive and tamoxifen-treated patients with BC (n = 210) 42, a significant correlation was observed between the reduced overall survival and high SETD1A mRNA expression levels (*P* = 0.0086; Figure 1B). Next, we determined the expression levels of SETD1A in TamR cells (Figure 1C). SETD1A protein levels were significantly higher in TamR cells than in the parent cells (MCF-7). In contrast, the expression levels of SETD1B and MLL1, another family of H3K4 methylation enzymes, were not significantly affected by tamoxifen resistance (Figure 1D). SETD1A protein levels were higher in tamoxifen-resistant T47D (TamR T47D) cells than in the parent cells (T47D), another ER-positive BC cell line (Figure 1E-F). Collectively, our results suggest that SETD1A plays a potential role in tamoxifen resistance in BC.

### Role of SETD1A in tamoxifen resistance in BC cells

**Figure 2:**
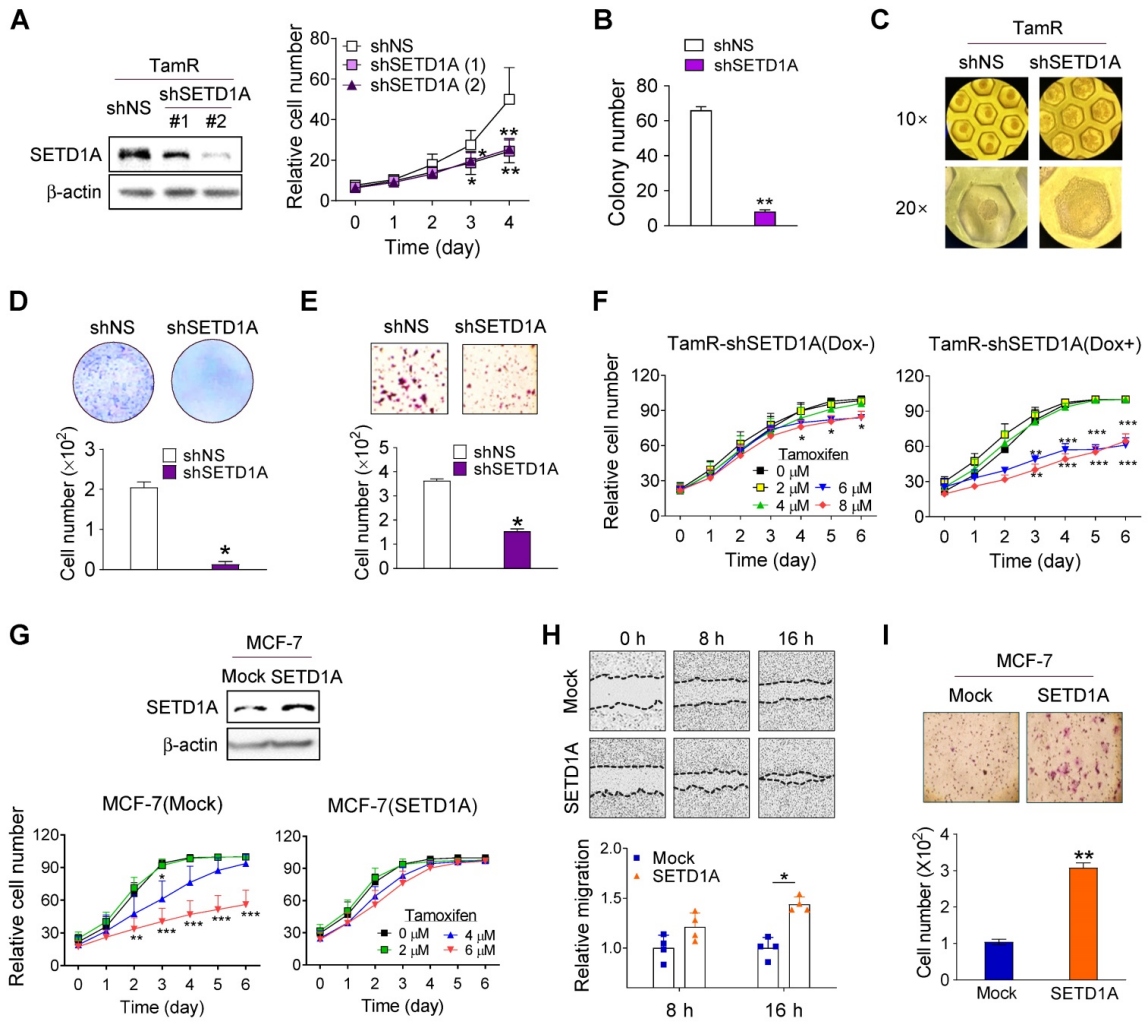
** Role of SETD1A in tamoxifen resistance in breast cancer cells. (A)** Cell proliferation in short hairpin RNA (shRNA)-silenced tamoxifen-resistant MCF-7 cells (TamR) was analyzed using a live cell imaging system. Each value represents the mean ± S.D. (n = 6). **(B)** Soft agar assay of TamR cells transfected with non-specific shRNA (shNS) or shRNA targeting mRNA of SETD1A (shSETD1A). Cells were plated on soft agar and cultured for 30 days. **(C)** The 3D-culture of TamR cells transfected with shNS or shSETD1A. Microwell culture dish was used for shRNA-silenced TamR spheroid culture for 24 h. **(D, E)** Transwell migration **(D)** and invasion **(E)** assays of SETD1A-depleted TamR cells. Data are expressed as the mean ± S.D. (n = 3). **(F)** Effect of SETD1A depletion in TamR cells on tamoxifen sensitivity. Control TamR cells (Dox-, left panel) and Dox-induced SETD1A knockdown TamR cells (right panel) were grown in the presence of tamoxifen, and cell proliferation was analyzed using a live cell imaging system. **(G)** Effect of SETD1A overexpression in MCF-7 cells on tamoxifen sensitivity. MCF-7 cells stably overexpressing SETD1A or control (Mock) were grown in the presence of tamoxifen, and cell proliferation was analyzed using a live cell imaging system. Each value represents the mean ± S.D. (n = 9). **(H)** Cell migration assay of SETD1A-overexpressing MCF-7 cells. Motility of MCF-7 cells stably overexpressing SETD1A or control (Mock) cells was examined after 8 and 16 h. Representative cell migration images are shown (top panel), and cell migration was quantified by measuring the distance between the opposing cell boundaries (bottom panel). Data are expressed as the mean ± S.D. (n = 3). **(I)** Invasion assay of SETD1A-overexpressing MCF-7 cells. Data are presented as the mean ± S.D. (n = 4). *P < 0.05; ** P < 0.01; *** P < 0.001.

**Figure 3:**
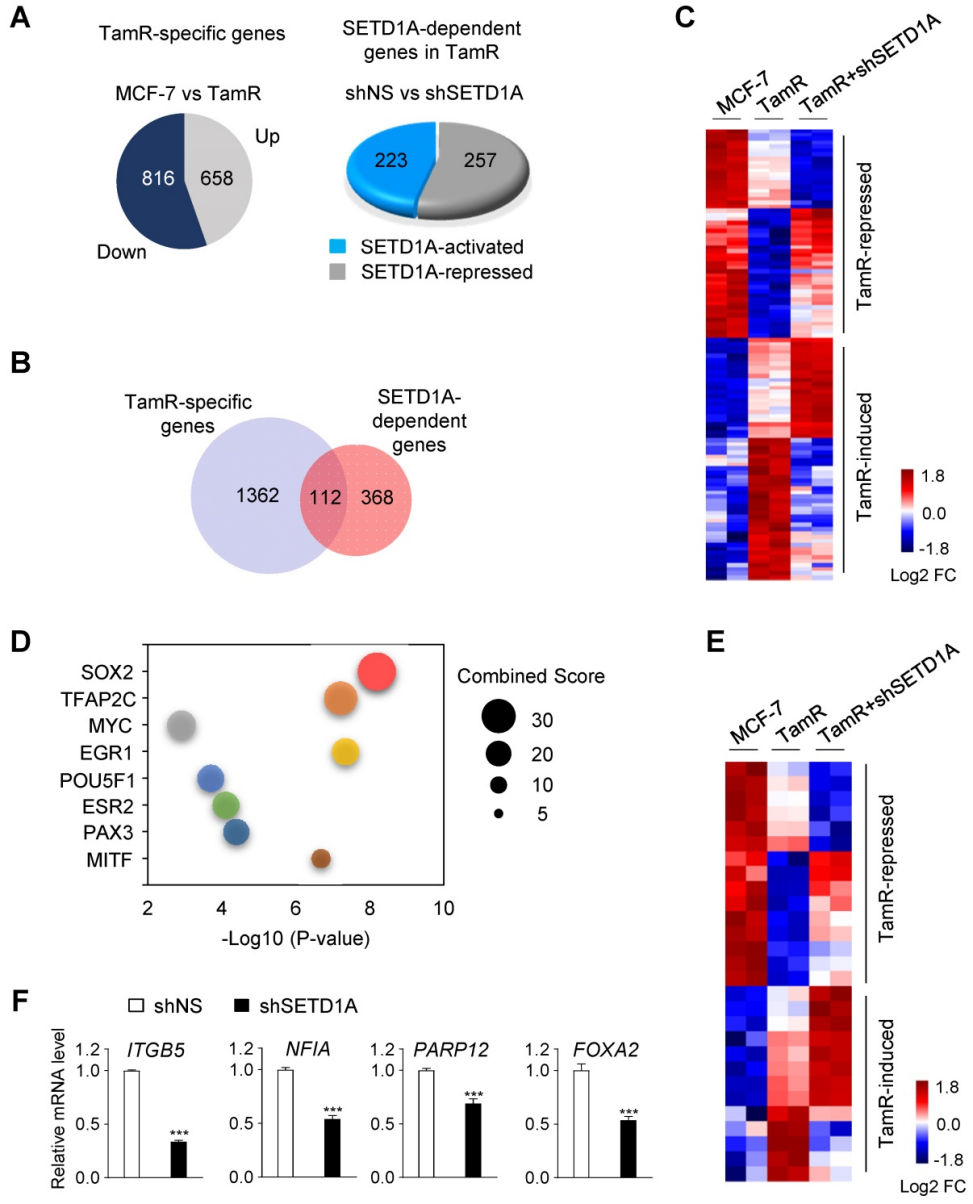
** Genome-wide analysis of SETD1A-dependent genes in MCF-7 and TamR cells. (A)** TamR-specific genes were identified based on RNA-sequencing (RNA-seq) results of MCF-7 and TamR cells (|FC| ≥ 1.5, *P* < 0.05). SETD1A-dependent genes in TamR cells were identified by performing RNA-seq analysis after SETD1A depletion by shRNA in TamR cells. **(B)** Venn diagram summarizing the RNA-seq results for TamR-specific gene set and SETD1A-dependent genes in TamR cells. **(C)** Heatmap generated via RNA-seq analysis of MCF-7 and TamR cells expressing either shNS or shSETD1A showing differential expression of SETD1A-dependent genes among TamR-specific genes. **(D)** Bubble chart of top 8 transcription factors predicted using Expression2Kinases analysis. Y-axis represents the transcription factor, X-axis represents the *P*-value, and the size of bubble represents the Combined Score. **(E)** Effect of SETD1A depletion on the expression levels of SOX2 target genes among TamR-specific genes. **(F)** RT-qPCR validation of the effect of SETD1A knockdown on SOX2 target genes in TamR cells. Data are presented as the mean ± S.D. (n = 4). *** P < 0.001.

Based on the finding that SETD1A protein levels were increased in TamR cells, we sought to investigate the specific function of SETD1A in TamR cells. Similar to our previously published results 39, short hairpin RNA (shRNA) knockdown of SETD1A resulted in the significant downregulation of TamR cell proliferation (Figure 2A). In addition, SETD1A-depleted TamR cells displayed significantly lower colony and spheroid formation than the controls in both soft agar (Figure 2B) and 3D cultures that more accurately reflected the *in vivo* environment (Figure 2C). Moreover, SETD1A depletion markedly inhibited the migration and invasion of TamR cells (Figure 2D-E). To explore the biological effects of SETD1A on tamoxifen resistance, we investigated the effects of SETD1A knockdown in TamR cells or SETD1A overexpression in MCF-7 cells on sensitivity to tamoxifen. Compared with the control group, SETD1A-depleted TamR cells exhibited restored tamoxifen sensitivity (Figure 2F), whereas SETD1A overexpression resulted in a decreased sensitivity of MCF-7 cells to tamoxifen (Figure 2G). In addition, SETD1A overexpression in MCF-7 cells enhanced their migration and invasion abilities (Figure 2H-I). These data indicate that SETD1A plays an important role in the development of TamR BC cells.

### SETD1A regulates the transcription of SOX2 target genes in tamoxifen-resistant cells

To investigate the mechanism of action of SETD1A in the development of tamoxifen resistance, a transcriptome analysis was conducted in MCF-7 and TamR cells. First, compared with MCF-7 cells, 1474 TamR-specific genes that were differentially expressed in TamR cells were identified (Figure 3A). Among these, 112 genes that were specifically affected by SETD1A knockdown were identified (Figure 3B-C). Next, we investigated the regulatory factors of these 112 genes using Expression2Kinase(X2K) 40. SOX2 was predicted to be a potential transcriptional regulator of these genes (Figure 3D). Indeed, the expression levels of many known SOX2 target genes were altered in TamR cells, and most of these genes were affected by the depletion of SETD1A (Figure 3E). The effect of SETD1A on the expression levels of SOX2 target genes in TamR cells was validated via RT-qPCR (Figure 3F). Notably, SOX2 is a well-known prognostic biomarker for tamoxifen resistance 26. Gene Ontology biological process analysis showed that the regulation of TamR-specific genes by SETD1A was enriched in tumor-associated pathways, such as apoptotic process, mitogen-activated protein kinase cascade, and cell migration (Figure S1). These pathway analyses support the hypothesis that SETD1A regulates the expression levels of SOX2 target genes in TamR BC cells.

### Correlation between SETD1A and SOX2 expression levels in tamoxifen-resistant cells

**Figure 4:**
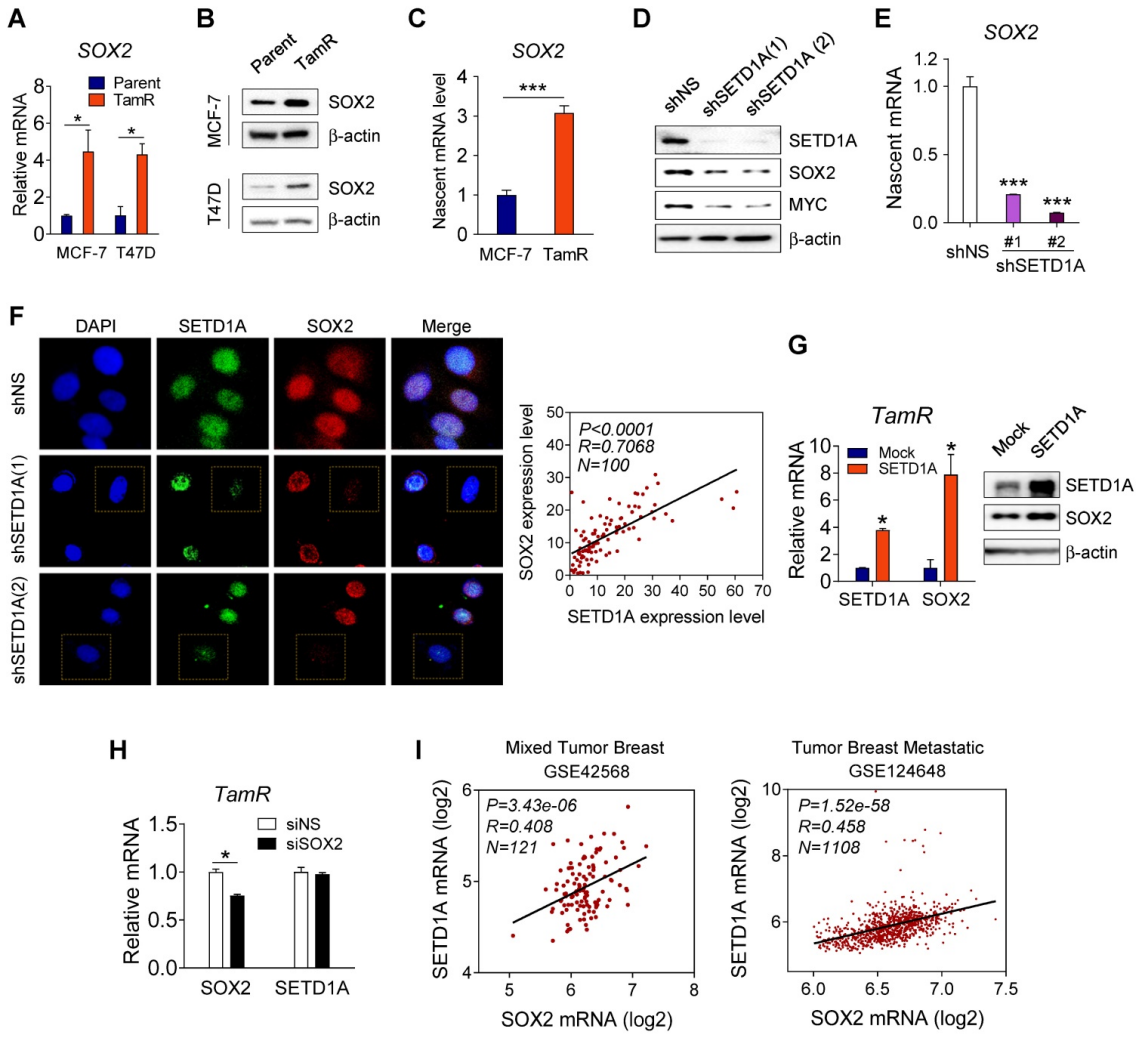
** Correlation between SETD1A and SOX2 expression levels in tamoxifen-resistant cells. (A, B)** Comparison of SETD1A expression levels in parent breast cancer cells and corresponding tamoxifen-resistant cells. Expression levels of SOX2 were measured via RT-qPCR and western immunoblotting in MCF-7 and T47D cells and tamoxifen-resistant cells (TamR MCF-7 and TamR T47D cells). Data are expressed as the mean ± S.D. (n = 3). **(C)** Analysis of nascent mRNA in MCF-7 and TamR cells. Newly synthesized RNA was labeled, and nascent SOX2 mRNA was analyzed via RT-qPCR. Levels of all nascent mRNAs were normalized to that of* GAPDH*. Data are expressed as the mean ± S.D. (n = 4). **(D)** Effect of SETD1A depletion using shRNA on SOX2 protein level in TamR cells. Extracts from the cells were analyzed for SETD1A, SOX2, MYC, and β-actin levels via western immunoblotting. **(E)** Analysis of nascent SOX2 mRNA levels in SETD1A-depleted TamR cells. Data are expressed as the mean ± S.D. (n = 3). **(F)** Immunofluorescence analysis of SETD1A (green) and SOX2 (red) in TamR cells transfected with shNS or shSETD1A. Plotted data show the intensity levels of SETD1A and SOX2 fluorescence (right panel). Pearson correlation analysis of SETD1A and SOX2 expression levels was conducted using GraphPad Software Prism v.8.0.2 (n = 100). **(G)** Effect of SETD1A overexpression on SOX2 levels in TamR cells measured via RT-qPCR and western immunoblotting. Data are expressed as the mean ± S.D. (n = 3). **(H)** Effect of SOX2 depletion on SETD1A expression levels in SOX2-depleted TamR cells measured via RT-qPCR. Data are presented as the mean ± S.D. (n = 4). **(I)** Correlation analysis of SETD1A and SOX2 expression levels in patients with breast cancer. Plotted data show log2 mRNA expression levels in GSE 42568 and GSE 124648 datasets. *P < 0.05; ** P < 0.01; *** P < 0.001.

The finding that SOX2 regulates the expression of SOX2 target genes in TamR cells led us to investigate whether SETD1A regulated the expression of SOX2. First, we observed that compared to the parent cells, SOX2 mRNA and protein levels were significantly increased in TamR BC cells (TamR MCF-7 and TamR T47D) (Figure 4A-B). A similar result was observed for nascent mRNA level of SOX2 (Figure 4C). Next, we investigated whether SETD1A regulated the *SOX2* gene transcription. Expression of a specific shRNA for SETD1A (shSETD1A) significantly reduced the SOX2 protein and nascent mRNA levels in TamR MCF-7 cells (Figure 4D-E). Immunofluorescence analysis of endogenous SETD1A and SOX2 showed that these proteins coexist in the nucleus. Specifically, the expression levels of SETD1A and SOX2 proteins were correlated in each cell; TamR cells exhibiting low levels of SETD1A exhibited low levels of SOX2 and *vice versa* (Figure 4F). In contrast, SETD1A overexpression in TamR and MCF-7 cells further increased SOX2 mRNA and protein levels (Figure 4G and Figure S2). However, downregulation of SOX2 did not affect the SETD1A mRNA levels (Figure 4H). Supporting the relevance of these findings, a positive correlation between SETD1A and SOX2 expression levels was observed in patients with BC (Figure 4I). Collectively, these results suggest that SETD1A acts as an upstream regulator of SOX2 transcription in TamR cells.

### SETD1A regulates the transcription of *SOX2*

**Figure 5:**
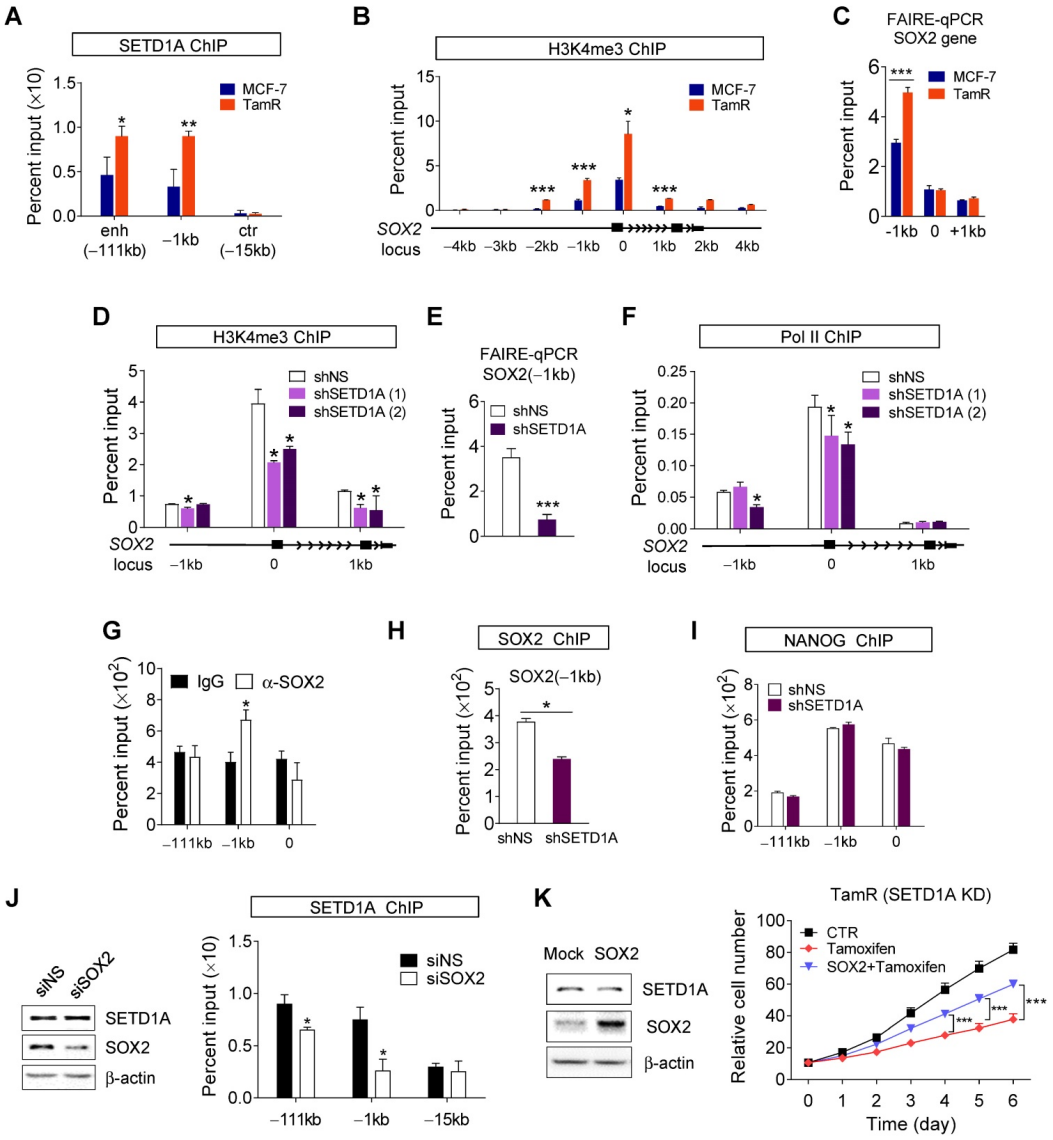
** SETD1A regulates the transcription of *SOX2* gene. (A, B)** SETD1A recruitment and histone H3K4me3 level at the *SOX2* gene in parent breast cancer and corresponding tamoxifen-resistant cells. Chromatin immunoprecipitation (ChIP) assay was performed in MCF-7 and TamR cells. Quantification of the indicated region of *SOX2* gene precipitated by anti-SETD1A or anti-H3K4me3 antibodies was performed via qPCR. Data are expressed as the mean ± S.D. (n = 3). **(C)** Chromatin accessibility at the *SOX2* locus assessed by formaldehyde-assisted isolation of regulatory elements (FAIRE)-qPCR analysis using chromatin samples from MCF-7 and TamR cells. Data are normalized to non-crosslinked genomic DNA for each primer pair. Data are presented as the mean ± S.D. (n = 3). **(D)** Role of SETD1A in H3K4me3 methylation at the *SOX2* locus. SETD1A levels in TamR cells were depleted using shRNA. Data are expressed as the mean ± S.D. (n = 3). **(E)** Effect of SETD1A on chromatin accessibility at *SOX2* gene promoter. FAIRE-qPCR analysis was performed at the promoter region (*-*1 kb) of *SOX2* gene in SETD1A-depleted TamR cells. Data are expressed as the mean ± S.D. (n = 3). **(F)** Effect of SETD1A on recruitment of RNA polymerase II to the *SOX2* gene promoter region in TamR cells. Quantification of the indicated region of *SOX2* gene precipitated by anti-SOX2 antibody was performed via qPCR. Data are expressed as the mean ± S.D. (n = 3) **(G)** Recruitment of SOX2 to the *SOX2* gene in TamR cells. **(H, I)** Effect of SETD1A depletion on the recruitment of SOX2 and NANOG to the *SOX2* gene in TamR cells. Data are presented as the mean ± S.D. (*n* = 3). **(J)** Effect of SOX2 depletion on the recruitment of SETD1A to the *SOX2* promoter or enhancer region in TamR cells. **(K)** Restoration of tamoxifen resistance by SOX2 overexpression in SETD1A-depleted TamR cells. Sensitivity to tamoxifen was measured after transfection of the control or SOX2-overexpressing plasmids in SETD1A-depleted TamR cells (tamoxifen vs SOX2 + tamoxifen). Increase in SOX2 protein levels by the SOX2 expression plasmid was measured by western blotting (left panel). Cell proliferation was analyzed using a live cell imaging system (right panel). Data are expressed as the mean ± S.D. (n = 3). *P < 0.05; **P < 0.01; ***P < 0.001.

To elucidate the basic mechanism by which SETD1A regulates SOX2 transcription, SETD1A recruitment to the *SOX2* gene in MCF-7 and TamR cells was investigated. ChIP analysis showed that the promoter (-1kb) and super-enhancer regions of the *SOX2* gene (-111 kb) were occupied by SETD1A, and SETD1A recruitment was higher in TamR cells than in MCF-7 cells (Figure 5A). ChIP assay using anti-H3K4me3 showed specific enrichment of H3K4me3 at the transcription start site (TSS) of *SOX2* gene. Importantly, we observed that H3K4me3 was higher in the promoter region of the TSS of *SOX2* gene in TamR cells than in MCF-7 cells (Figure 5B). These results are consistent with the finding of high recruitment of H3K4 methylation enzyme SETD1A to the *SOX2* gene in TamR cells. The correlation between H3K4 methylation and chromatin accessibility is well documented. Consistently, chromatin accessibility of the promoter region of the *SOX2* gene was increased in TamR cells (Figure 5C). However, SETD1A knockdown using shRNA reduced H3K4me3 in the promoter region (Figure 5D) and decreased chromatin accessibility of the promoter region (Figure 5E) of the *SOX2* gene, eventually leading to reduced recruitment of RNA polymerase II (Pol II; Figure 5F).

ChIP-seq analysis of immortalized multipotent otic progenitor cells revealed that SOX2 acts as a transcription factor that regulates the expression of *SOX2* gene 44. Similar results were observed in our study using TamR BC cells. SOX2 protein was recruited to the promoter region of *SOX2* gene (Figure 5G). This recruitment was reduced by SETD1A knockdown, which may be due to a decrease in SOX2 protein levels after SETD1A knockdown (Figure 5H). In contrast, recruitment of NANOG, another transcription factor regulating SOX2 expression, to the *SOX2* gene was not altered by SETD1A knockdown (Figure 5I). Depletion of SOX2 in TamR cells resulted in a reduction in SETD1A recruitment to the promoter and enhancer regions of the *SOX2* gene without affecting SETD1A protein levels (Figure 5J). These results indicate that SETD1A binds to the promoter and enhancer regions of the *SOX2* gene in SOX2-dependent manner to induce H3K4me3 at the promoter and TSS sites and to activate SOX2 transcription by maintaining the optimal chromatin structure required for transcription. To validate these results, we examined the role of SETD1A in the expression of two other SOX2 target genes, *MYC* and *BMP7*. Knockdown of SOX2 reduced mRNA of MYC in TamR cells (Figure S3A). SOX2 and SETD1A were recruited to the promoter and TSS regions of the *MYC* gene (Figure S3B-C). Trimethylation of histone H3K4 in *MYC* gene was SETD1A-dependent, and SETD1A regulated chromatin accessibility of MYC gene (Figure S3D-E). SETD1A protein was recruited to the MYC gene in a SOX2- dependent manner, and SETD1A knockdown reduced the nascent mRNA and protein levels of MYC in TamR cells (Figure S3F-G and Figure 4D). Additionally, SETD1A was recruited to the *BMP7* gene, regulated H3K4 trimethylation and chromatin remodeling, eventually affecting the recruitment of Pol II to the *BMP7* gene (Figure S4). These results indicate that SETD1A binds in SOX2-dependent manner to the promoter and enhancer regions of SOX2 target genes, including the *SOX2* gene itself, to induce H3K4me3 at the promoter and TSS sites, and to activate SOX2 transcription by maintaining the optimal chromatin structure required for transcription. Consistently, overexpression of SOX2 in SETD1A knockdown TamR cells restored tamoxifen resistance (Figure 5K), indicating that SETD1A promoted tamoxifen resistance via SOX2 signaling.

### SETD1A interacts with SOX2 via the Win motif

**Figure 6:**
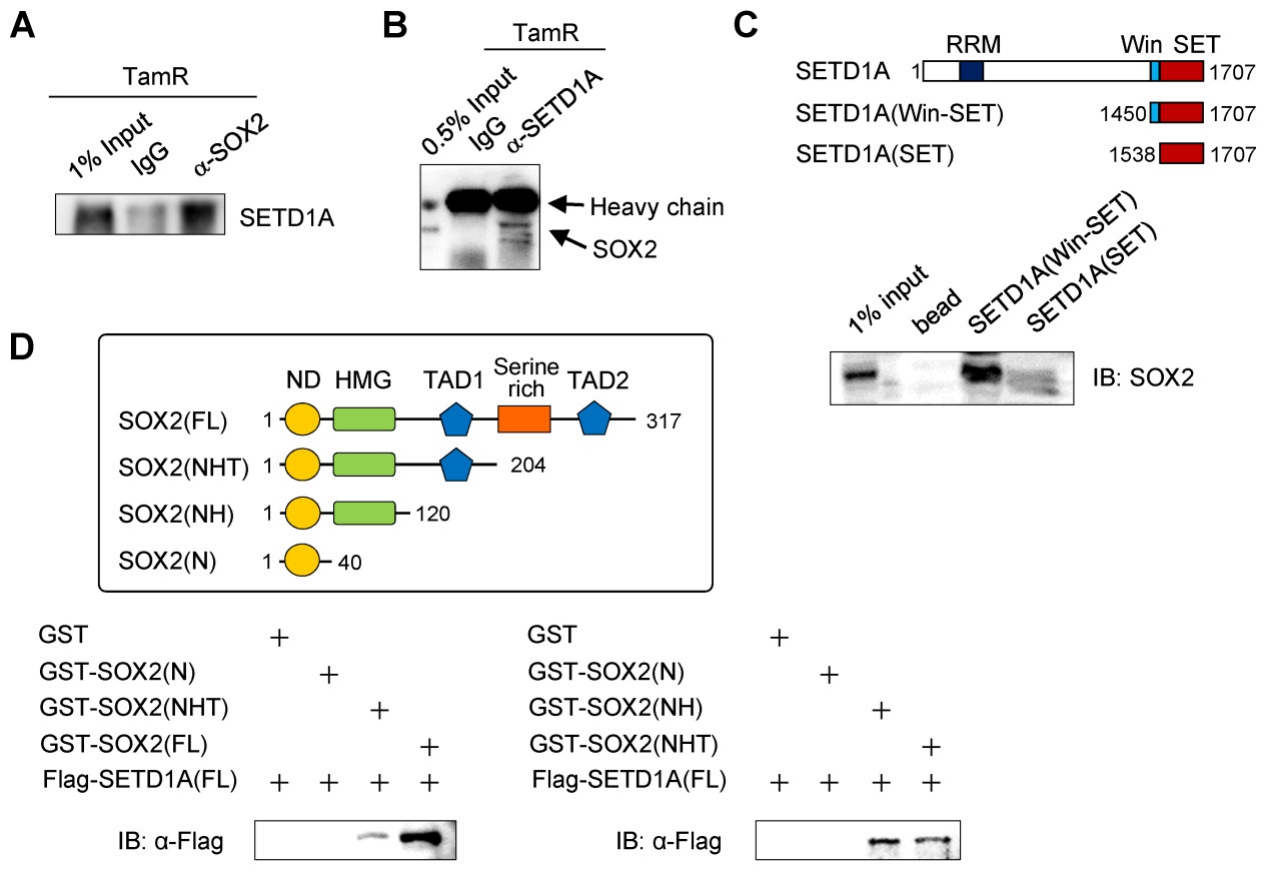
** Binding of SETD1A to SOX2 via the Win motif. (A, B)** The interaction between SOX2 and SETD1A in TamR cells was determined by co-immunoprecipitation using anti-SOX2 or anti-SETD1A antibody followed by western immunoblotting of SETD1A or SOX2. **(C)** Direct binding assay using recombinant SET domain of SETD1A. Two types of His-tagged SET domains of SETD1A protein with (Win-SET) or without (SET) the Win motif were expressed in *E. coli*. After incubation of these bead-bound SET domains and TamR cell lysates immobilized in Ni-NTA beads, SOX2 protein bound to SETD1A was identified as an anti-SOX2 antibody. **(D)** Mapping study of SOX2 to identify SETD1A binding region. GST-tagged truncation mutants of SOX2 were expressed in *E. coli* and incubated with FLAG-tagged full-length SETD1A expressed using *in vitro* transcription and translation system. SETD1A bound to the SOX2 fragments was measured using the anti-FLAG antibody.

Our previous results suggest that SETD1A might associate with SOX2 at SOX2 target gene sites, including the *SOX2* gene itself. In addition, the binding of SETD1A to the SOX2 target gene site is SOX2-dependent. Therefore, we investigated the direct interaction between SETD1A and SOX2 (Figure 6 and Figure S5). First, we observed that endogenous SETD1A was associated with SOX2 in TamR cells using a co-immunoprecipitation assay (Figure 6A-B). Next, we investigated whether SOX2 and SETD1A bind directly and identified their binding sites. *In vitro* binding assay revealed that the Win motif of SETD1A directly binds to SOX2 (Figure 6C). Previous studies have revealed direct binding of SOX2 and Ash2L subunits of the SET1/MLL complex 45, and it has been reported that the Win motif of SETD1A provides a binding site for various proteins, including WDR5 46. In this study, the Win motif played an important role in the interaction between SETD1A and SOX2 (Figure 6C). In addition, in vitro protein-binding experiments using various truncated forms of SOX2 have revealed that the HMG domain (a.a. 41-120) of SOX2 directly binds to SETD1A. The presence of the C-terminal region of SOX2 (serine-rich domain and TAD2, a.a. 205-317) further increased binding to SETD1A (Figure 6D).

### Role of SETD1A in tamoxifen resistance of cancer stem cells

**Figure 7:**
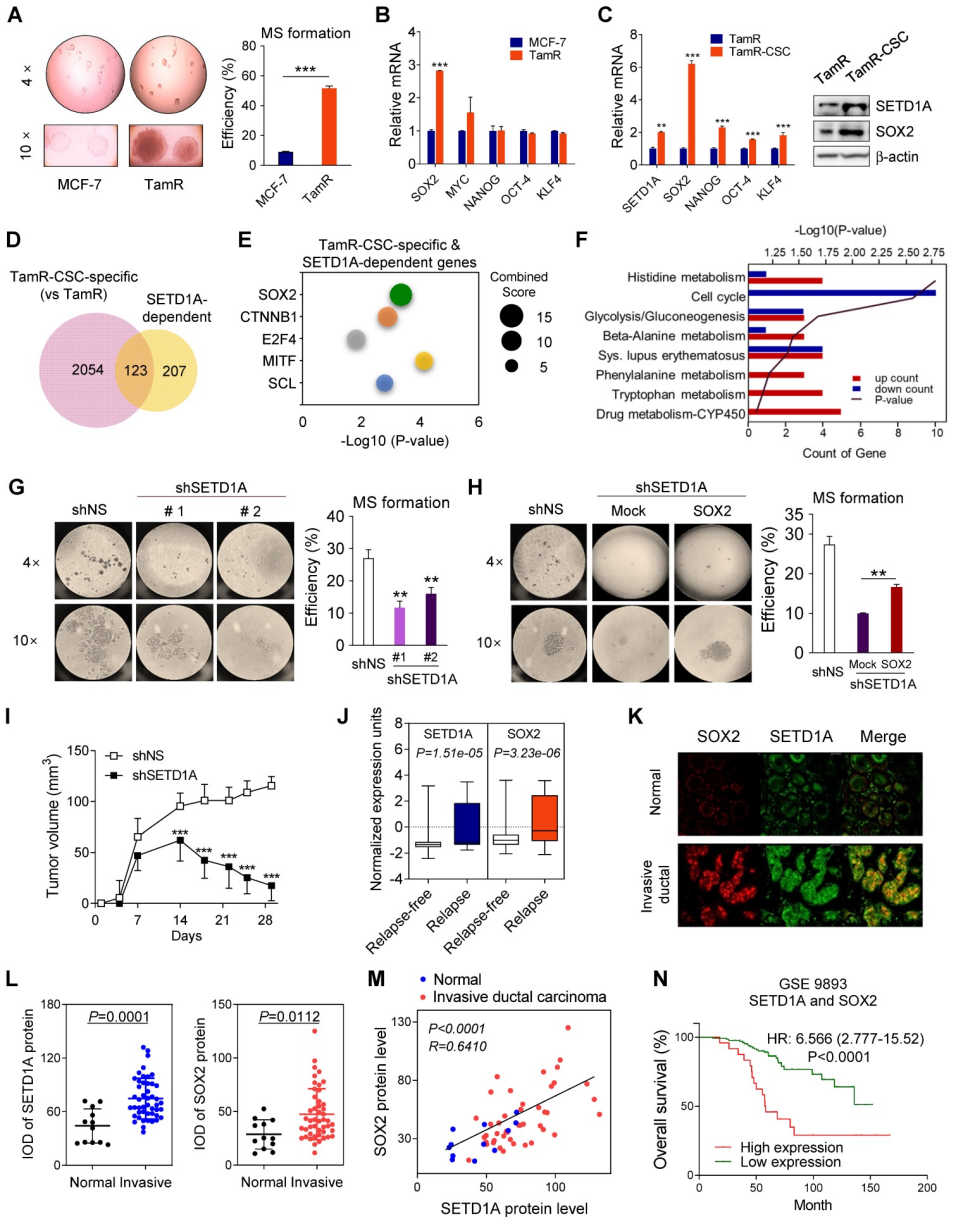
** The role of SETD1A in cancer stem cells in TamR. (A)** Mammosphere (MS) formation assay of MCF-7 and TamR cells. The efficiency of mammosphere formation was calculated by dividing the number of mammospheres formed by the number of cells plated. Data are expressed as the mean ± S.D. (n = 3). **(B)** Expression levels of SETD1A and stem cell factors, including* SOX2, MYC, NANOG, OCT-4,* and* KLF4* in MCF-7 and TamR cells. Data are expressed as the mean ± S.D. (n = 3). **(C)** Increased expression levels of stem cell factors in cancer stem cells isolated from TamR cells (TamR-CSC) compared to that in TamR cells. Data are expressed as the mean ± S.D. (n = 3). **(D)** Venn diagram summarizing the RNA-seq results for cancer stem cell-specific genes (*versus* TamR) and SETD1A-dependent genes in cancer stem cells (|FC| ≥ 1.5, *P* < 0.05). **(E)** Bubble chart of top 5 transcription factors predicted using SETD1A-dependent genes in cancer stem cells Expression2Kinases suite analysis. Y-axis represents the transcription factor, X-axis represents the *P-*value, and the size of bubble represents the Combined Score. **(F)** Gene Ontology (GO) biological process analysis of SETD1A-dependent genes among cancer stem cell-specific genes. Statistically significant pathways (*P* < 0.05) are listed. **(G)** Mammosphere formation assay of SETD1A-depleted TamR cells. Data are expressed as the mean ± S.D. (n = 3). **(H)** Effect of SOX2 overexpression on tamoxifen-resistance in SETD1A-depleted TamR cells. The formation of mammosphere was measured after transfection of the control or SOX2-overexpressing plasmids in SETD1A-depleted TamR cells (mock vs SOX2). **(I)** SETD1A regulates the *in vivo* proliferation of TamR cells in the mouse xenograft model. TamR cells (shNS or shSETD1A) were injected subcutaneously into nude mice. Tumor volumes are shown as the mean ± S.D. (n = 10). ***P < 0.001. **(J)** The mRNA levels of SETD1A and SOX2 in ERα-positive and tamoxifen-treated patients with breast cancer (GSE9893). The 25th-75th percentiles are indicated by the closed box. **(K)** Representative image of SETD1A and SOX2 protein level in patients with breast cancer. Invasive: invasive ductal carcinoma; Normal: adjacent normal tissue. **(L)** Comparison of SETD1A and SOX2 protein level in normal breast tissue and invasive ductal carcinoma tissues. SETD1A and SOX2 protein expression levels were determined via immunofluorescence staining in human breast cancer tissue microarrays containing the normal breast tissues (n = 12) and invasive ductal carcinoma tissues (n = 47). SETD1A and SOX2 protein levels in individual tissue samples were presented as average integrated optical density (IOD). **(M)** Correlation between SOX2 and SETD1A protein levels in breast cancer tissue. Plotted data show the protein levels of SETD1A and SOX2 in normal breast tissues (n = 12) and invasive ductal carcinoma tissues (n = 47). Pearson correlation analysis of *SETD1A* and *SOX2* expression levels was conducted using the GraphPad Software Prism V.8.0.2. **(N)** Kaplan-Meier overall survival curves show the prognostic ability of SETD1A and SOX2 signatures. The high-expressing group was defined as having both SETD1A and SOX2 levels higher than the median of all patients that participated in the study, while the remaining patients were in the low-expressing group.

SOX2, an embryonic stem cell marker, along with NANOG and OCT-4 is highly expressed in BC cells 47. Therefore, we investigated mammosphere formation during the development of tamoxifen resistance. First, TamR cells formed significantly more mammospheres than control cells (Figure 7A), suggesting an increased self-renewal capacity. Second, among the stem cell factors reported to be involved in maintaining the self-renewal capacity of epithelial stem cells (ESCs), a significant difference between TamR and MCF-7 cells was observed only with respect to SOX2 expression (Figure 7B). However, in CSC isolated from TamR cells (TamR-CSC), the expression levels of all four stem cell factors, SOX2, OCT-4, NANOG, and KLF4, were significantly increased. However, SOX2 expression was most significantly increased (Figure 7C), consistent with the results of previous studies revealing the involvement of SOX2 in tamoxifen resistance 26. This suggests that the increased expression of SOX2 by SETD1A plays an important role in the development of tamoxifen-induced resistance and stem/progenitor cell population formation in BC cells.

Our results show that SETD1A regulates the expression levels of SOX2 in TamR cells, suggesting that SETD1A may be involved in the self-renewal of BC stem cells (BCSCs). First, to investigate the genome-wide effects of SETD1A in cancer stem cell-specific gene expression, RNA-seq analysis was conducted in TamR cells and TamR-CSCs. Compared with TamR cells, 2177 genes were differentially expressed in TamR-CSCs; among them, 123 genes regulated by SETD1A were identified (Figure 7D and Figure S6). Next, by predicting the potential upstream regulators of these 123 genes, we confirmed that SOX2 was the most significant (*P* = 4.61E-04) protein among the upstream transcription factors of TamR-CSC-specific and SETD1A-dependent genes (Figure 7E). Indeed, the expression levels of many known SOX2 target genes were affected by the depletion of SETD1A in TamR-CSCs (Figure S6). Additionally, depletion of SETD1A resulted in the downregulation of all DNA replication and ubiquitination-related genes in TamR-CSCs, which participate in the maintenance of CSC stemness 48, 49 (Figure 7F). Next, we examined the role of SETD1A in the self-renewal of TamR-CSCs. Downregulation of SETD1A expression significantly reduced the mammosphere formation in TamR cells (Figure 7G). However, this effect was reversed by SOX2 overexpression (Figure 7H). Additionally, SETD1A-depleted TamR cells showed significantly decreased growth compared with the control group in a mouse xenograft model (Figure 7I). Finally, we determined the clinical relevance of SETD1A-SOX2 expression in the prognosis in TamR BC. In the transcriptome analysis of patients with ER-positive BC receiving tamoxifen, SETD1A and SOX2 transcriptional levels were significantly increased in tamoxifen-resistant patients than in the tamoxifen-sensitive patients (Figure 7J). Additionally, the protein levels of SETD1A and SOX2 were significantly higher in invasive ductal carcinoma tissues than in the normal breast tissues (Figure 7K-L). Importantly, a positive correlation between SETD1A and SOX2 protein levels was observed in patients with BC (Figure 7M), supporting our findings regarding the mRNA levels in TamR cells and patients with BC (Figure 4). Higher levels of both SOX2 and SETD1A were significantly associated with overall survival rates (Figure 7N)**.** These results indicate that the BCSC-specific target gene regulation mechanism of the SETD1A-SOX2 axis plays an important role in the development of tamoxifen resistance and recurrence in patients via the formation and maintenance of BCSCs.

## Discussion

Endocrine therapy can significantly reduce the mortality and recurrence rates by inhibiting ER signaling, thereby improving the survival rate in patients with ER-positive BC 50, 51. However, more than 30% of ER-positive BC cases are intrinsically resistant to hormone therapy, and in a certain proportion of patients with BC, resistance to long-term endocrine therapy is inevitable (acquired resistance) 52, 53. Currently, the treatment strategy for endocrine resistance involves the combination of hormone endocrine therapy with molecular targeting drugs such as those targeting mammalian target of rapamycin, cyclin-dependent kinase, or EGFR (www.clinicaltrials.gov) 54. However, the identification of novel therapeutic targets or more specific biomarkers is necessary for the successful treatment of BC refractory to endocrine therapy. Recent studies have shown that epigenetic mechanisms regulate the growth of CSC-like cell subsets, resulting in the resistance to anticancer drugs 55. HDACs required for maintaining CSCs, are found overexpressed in CSCs. The HDAC inhibitor, trichostatin A, was used to preferentially target CSCs 56. Additionally, breast CSCs have a unique miRNA expression profile 57, 58; for example, miRNA-200 targeting the CSC self-renewal factor BMI1 is downregulated in mammary CSCs 59.

Our study presents the clinical significance and mechanism of action of SETD1A, a histone H3K4-specific methyltransferase, in the development of tamoxifen resistance in BC. Data analysis of patients receiving tamoxifen monotherapy for more than five years revealed that SETD1A was strongly correlated with poor prognosis, and depletion of SETD1A in TamR BC cells led to alterations in the proliferation, migration, and invasion of these cells. We demonstrated that SETD1A plays an important role in TamR cell colonization and spheroid formation under both soft agar and 3D culture conditions. We also showed that SETD1A-depleted TamR cells exhibited significantly reduced tumor growth in a mouse xenograft model. Importantly, depletion of SETD1A in TamR cells restored their sensitivity to tamoxifen. Moreover, overexpression of SETD1A in MCF-7 cells led to the development of tamoxifen resistance, indicating that SETD1A is a key molecule that promotes tamoxifen resistance in BC cells. SETD1A directly binds to SOX2 and regulates the transcription of SOX2 target genes, including the *SOX2* gene itself. As SOX2 is involved in maintaining stem cells, our results indicate that SETD1A plays an important role in the self-renewal of CSCs by regulating SOX2.

According to the cancer stem cell theory, a small number of CSCs may remain dormant following conventional cancer therapy, and during tumor remission, they regenerate to cause recurrent cancer, which has profound implications for cancer therapy. Similarly, the increase in SETD1A expression in tamoxifen-resistant cells seems to be the result of the dominant survival of resistant clones, CSCs, expressing high levels of SETD1A and SOX2 due to intra-tumor heterogeneity in primary BC. A previous report indicated that SOX2 was overexpressed in TamR cells, which conferred the stem cell-like and resistant phenotypes to BC cells 26. SETD1A is involved in cell proliferation in various cancers, including lung cancer, colorectal cancer, hepatocellular carcinoma, and leukemia, and in the development of resistance to anticancer drugs 60-67.

Moreover, SETD1A is a key co-activator required for the transcription of OCT-4-mediated genes in ESCs 68. Our study demonstrated the interaction of SETD1A with SOX2, and SETD1A overexpression during the development of tamoxifen resistance in ER-positive BC cells. SETD1A expression levels were higher in TamR-CSCs than in TamR cells grown in adherent cultures, and depletion of SETD1A significantly reduced the mammosphere formation capacity of TamR cells. SETD1A knockdown resulted in changes in the expression levels of several genes, including those associated with CSCs. These results suggest that SETD1A plays an important role in the self-renewal of CSCs in TamR BC. Further research is needed to determine whether the origin of TamR-CSCs in TamR cells is a product of de novo resistance or newly acquired mutations during tamoxifen treatment. If the mutation is newly acquired, BC cells may adapt to anticancer treatments via epigenetic modifications. A recent report that anticancer drug resistance can be caused not only by the survival of the fittest mutation, but also by the adaptation of tumor cells to the environment via non-genetic (epigenetic) heterogeneity further supports the above hypothesis 37, 69. Collectively, our results suggest that SETD1A is a key regulator of SOX2 in BC cells and demonstrated the clinical significance of SETD1A in patients with BC that were treated with tamoxifen, indicating that SETD1A is a potential therapeutic target for the treatment of tamoxifen resistance.

## Supplementary Material

Supplementary methods, figures and tables.

## Abbreviations

AUC: area under the curve
BC: breast cancer
ChIP: chromatin immunoprecipitation
CoIP: co-immunoprecipitation
CSC: cancer stem cell
ER: estrogen receptor
FAIRE: formaldehyde-assisted isolation of regulatory elements
GST: glutathione S-transferase
HMG: high mobility group
4-HT: 4-hydroxytamoxifen
ITGB5: integrin subunit beta 5
KLF4: Kruppel-like factor 4
MLL1: mixed-lineage leukemia 1
NFIA: nuclear factor I A
OCT-4: octamer-binding transcription factor 4
Pol II: RNA polymerase II
SERM: selective estrogen receptor modulator
SETD1A: SET domain containing 1A
SETD1B: SET domain containing 1B
SOX2: SRY-box transcription factor 2
TamR: tamoxifen-resistant
TSS: transcription start site
