# Supplementary material for: SETD1A-SOX2 axis is involved in tamoxifen resistance in estrogen receptor α-positive breast cancer cells
Source: Theranostics. 2022 Jul 18;12(13):5761–75. doi: 10.7150/thno.72599 (PMC9373809; doi:10.7150/thno.72599)
Supplement: Supplementary file 1 — Supplementary methods, figures and tables. [file thnov12p5761s1.pdf]

Supplementary Materials

SETD1A-SOX2 axis is involved in tamoxifen resistance in estrogen receptor α-positive breast cancer cells

Ming Li Jin, Liu Yang, Kwang Won Jeong*

Supplementary Methods

Cell proliferation assay

Cells seeded in a 24-well plate (1×10^4 cells/well) plate and incubated at 37 °C with 5% CO2 were transferred to IncuCyte® ZOOM system (Essen Bioscience, Ann Arbor, MI, USA), which allowed automated live cell analysis within incubator. Images were captured at 4-h intervals using IncuCyte ZOOM software (version 2013B).

Soft agar assay and 3D cell culture

For soft agar assays, TamR cells expressing either shNS or shSETD1A were plated in DMEM and agarose (0.8 % top agarose and 1 % basic agarose). After 30 days of culture, colonies were stained with crystal violet and observed under a microscope to measure the size and number of each colony. For 3D spheroid culture, Prosys ® StemFit 3D (SF, Prodizen Inc., Seoul, Korea) was used. TamR cells expressing either shNS or shSETD1A were plated at a density of 1.0×10^6 cells/well. After incubation for 24 h, the aggregated cells were observed under a microscope.

Cell migration and invasion assay

Migration assay was conducted using a 24-well Transwell (Costar 3422; Corning Inc., Corning, NY, USA). Invasion assay was performed using BioCoat™ Matrigel® Invasion Chamber (354480, Corning Inc., Corning, NY, USA). Briefly, after transfection, 100 μL of serum-free DMEM and 200 μL of TamR cells (1.5×10^5 cells/mL) suspended in serum-free DMEM were added to the upper chamber. The cells were allowed to migrate for 24 h or invade for 48 h in the lower chamber, which contained 750 μL of the medium supplemented with 10 % FBS as a chemoattractant. Then, the cells were stained with 0.1 % crystal violet for 15 min, and washed with PBS. The cells remained in the upper chamber were removed with cotton wool, whereas the cells that had migrated or invaded were imaged with Nikon TS100 stereomicroscope coupled to Canon G10/G11 camera (Canon, Tokyo, Japan). Each insert captures at least three random field images, quantified with the ImageJ program.

Xenograft murine model

TamR cells (3×10^6 cells) expressing shNS or shSETD1A were suspended in Matrigel/PBS (100 μl) and injected subcutaneously into 6-week-old female BALb/c nu/nu mice (Orient Bio, Korea). The animals were randomly assigned to separate experimental groups (n = 10). No samples or animals were excluded from the analysis. The investigators were not blinded to group allocation. This study was conducted under ethical approval from the Institutional Animal Ethics Committee of Keyfron Bio Co., Ltd. (Cheongju, Korea)

RNA interference and RT-qPCR

Transfection of siRNA was performed using Oligofectamine (Invitrogen, Carlsbad, CA, USA) according to the manufacturer's protocol. The sequences of siRNAs and shRNAs are listed in Table S1. TamR cells expressing control shRNA or shRNA targeting SETD1A were generated *via* lentiviral transfection (Sigma-Aldrich, St. Louis, MO, USA). Inducible human SETD1A shRNA system was generated using mCMV-TurboGFP human SETD1A shRNA (Dharmacon, Lafayette, CO, USA). shRNA expression was induced through the addition of doxycycline (Dox, 0.5 μg/mL) for subsequent assay. Total RNA was isolated from TamR cells using TRIzol reagent (Invitrogen, Carlsbad, CA, USA), and cDNA was synthesized using iScript (Bio-Rad Laboratories, Hercules, CA, USA). RNA concentration was assessed spectrophotometrically using NanoDrop 2000 spectrophotometer (Thermo Fisher Scientific, Waltham, MA, USA). The reverse transcription product (2 μL) was used for real-time qPCR analysis using LightCycler 480 II instrument (Roche, Indianapolis, IN, USA) with the primers listed in Table S2. Relative gene expression levels were normalized to 18S rRNA levels.

Immunoblotting and immunocytochemistry

Cells were fixed with paraformaldehyde (4 %), and permeabilized with phosphate buffered saline containing 0.1 % TritonX-100, followed by blocking with PBST (0.1 % Tween 20) supplemented with 1 % bovine serum albumin; cells were then incubated overnight at 4 °C with rabbit anti-SETD1A antibody (Bethyl Laboratories, Montgomery, AL, USA) or mouse anti-SOX2 antibody (Invitrogen, Carlsbad, CA, USA), followed by the secondary antibodies such as anti-rabbit DyLight

488 and anti-mouse DyLight 594 (Vector Laboratories, Burlingame, CA, USA). Image was visualized using Nikon confocal microscope (Nikon, Tokyo, Japan). Western blotting was performed as previously described [1]. The antibodies used for western blotting are listed in Table S3.

Chromatin immunoprecipitation assay

Chromatin immunoprecipitation (ChIP) assay was performed according to a previously described protocol [2]. Briefly, after reaching approximately 90 % confluence, shRNA-transfected TamR cells were cross-linked with formaldehyde, and extracts were prepared. The sonicated chromatin solutions were immunoprecipitated by overnight incubation at 4 °C with anti-SETD1A (Bethyl Laboratories, Montgomery, AL, USA), anti-SOX2 (Invitrogen), anti-Pol II (Millipore, Massachusetts, USA), and anti-H3K4me3 (Active Motif, Carlsbad, CA, USA) antibodies. Results are expressed as the percentage of input chromatin (before immunoprecipitation). The primer sequences used for the ChIP assay are listed in Table S2.

FAIRE-qPCR

FAIRE-qPCR was performed as previously described [2]. Briefly, after reaching approximately 90% confluence, shRNA-transfected TamR cells were cross-linked with formaldehyde, and extracts were prepared. Results are expressed as the percentage of input chromatin (input DNA). The primer sequences used for FAIRE-qPCR are listed in Table S2.

Supplementary References

1. Jeong KW, Kim K, Situ AJ, Ulmer TS, An W, Stallcup MR. Recognition of enhancer element-specific histone methylation by TIP60 in transcriptional activation. Nat Struct Mol Biol. 2011; 18: 1358-65.

2. Jeong KW, Andreu-Vieyra C, You JS, Jones PA, Stallcup MR. Establishment of active chromatin structure at enhancer elements by mixed-lineage leukemia 1 to initiate estrogen-dependent gene expression. Nucleic Acids Res. 2014; 42: 2245-56.

Supplementary Figures

Figure S1

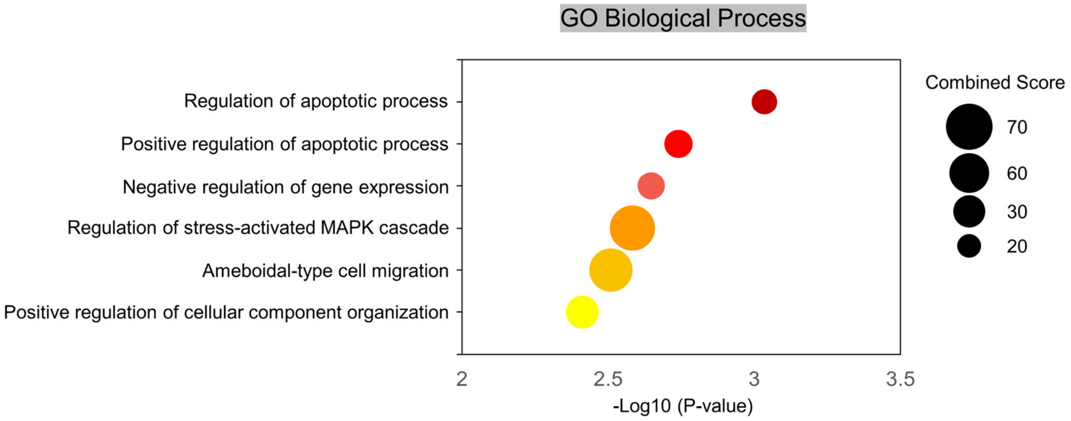

Figure S1. The functional role of SETD1A-dependant gene in TamR cells. Bubble chart of potential signaling pathways generated by SETD1A-dependant genes among TamR-specific genes. Pathway analysis was performed using the GO Biological Process database.

Figure S2

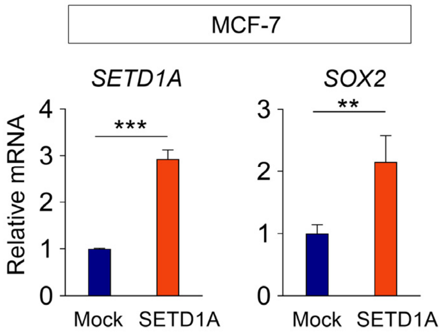

Figure S2. Effect of SETD1A overexpression on SOX2 mRNA level in MCF-7 cells. The expression of SOX2 were measured by RT-qPCR in Mock and SETD1A overexpressed MCF-7 cells. Data are expressed as the mean ± S.D. (n = 3) ***P*<0.01; ****P*<0.001.

Figure S3

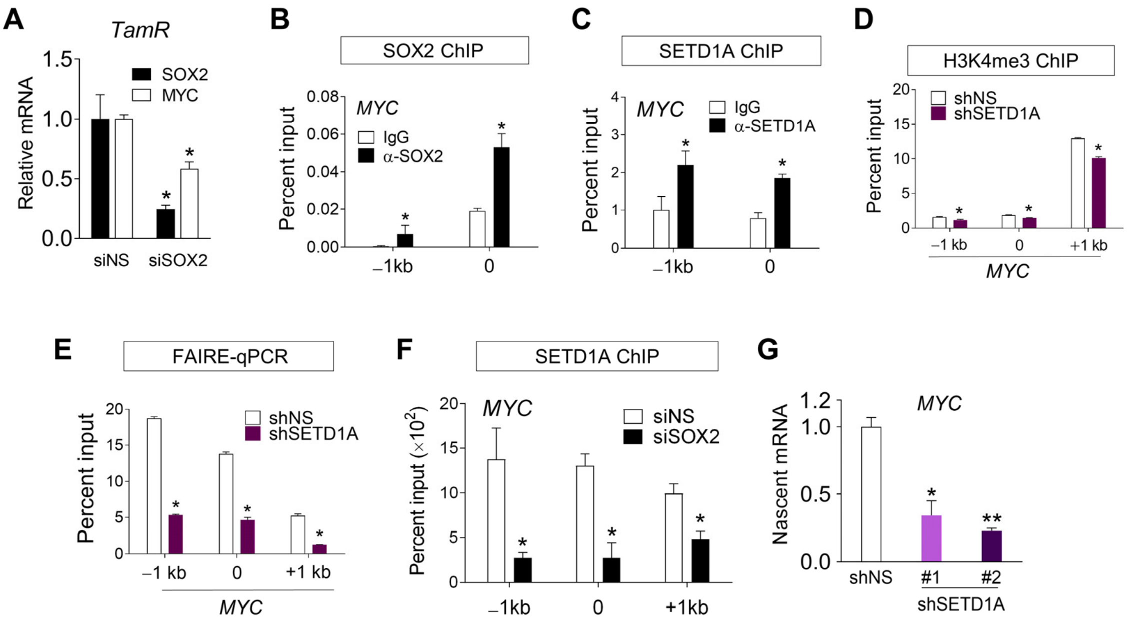

Figure S3. SETD1A regulates the transcription of the *MYC* gene. (A) The expression of *MYC* and *SOX2* was measured by RT-qPCR in SOX2-depleted Tam-R cells. Data are expressed as the mean ± S.D. (n = 3). **(B, C)** ChIP assays were performed in TamR cells. The amount of indicated region of the *MYC* gene precipitated by anti-SOX2 or anti-SETD1A antibody was determined by qPCR. Data are expressed as the mean ± S.D. (n = 3). **(D)** Role of SETD1A in H3K4me3 methylation at the *MYC* locus. SETD1A in TamR cells was depleted using shRNA. Data are expressed as the mean ± S.D. (n = 3). **(E)** Effect of SETD1A on the chromatin accessibility at *MYC* gene. FAIRE-qPCR analysis was performed at the *MYC* locus in SETD1A-depleted TamR cells. Data are expressed as the mean ± S.D. (n = 3). **(F)** SETD1A ChIP assays were performed in SOX2-depleted TamR cells. The amount of the indicated region of the *MYC* gene precipitated by the anti-SETD1A antibody was determined by qPCR. Data are expressed as the mean ± S.D. (*n* = 3). **(G)** Analysis of nascent *MYC* mRNA level in SETD1A-depleted TamR cells. Data are expressed as the mean ± S.D. (n = 3). *P<0.05; **P<0.01.

Figure S4

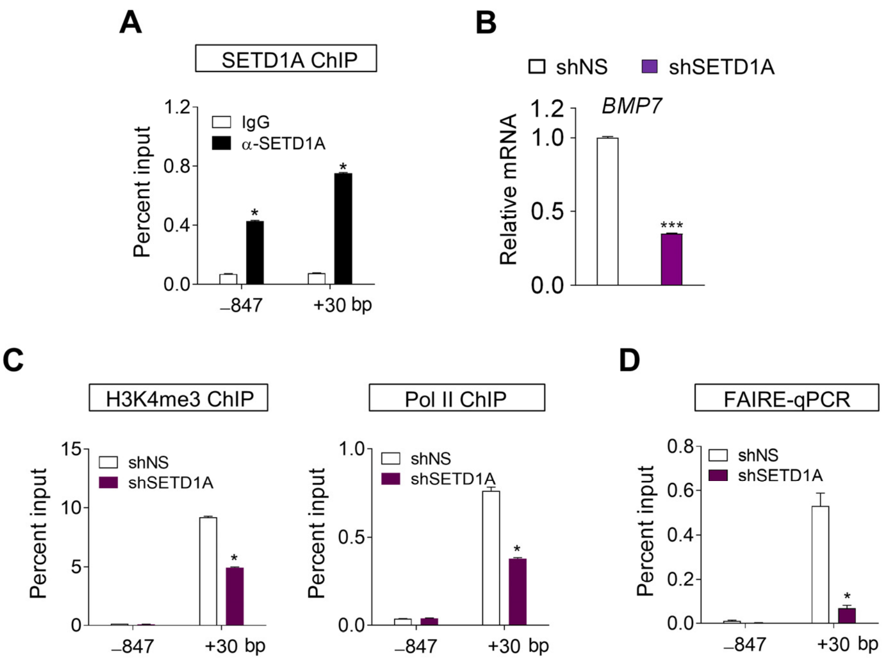

Figure S4. SETD1A regulates the transcription of the *BMP7* gene. (A) ChIP assays were performed in TamR cells. The amount of the indicated region of the *BMP7* gene precipitated by anti-SETD1A antibody. **(B)** The expression of *BMP7* was measured by RT-qPCR in SETD1A-depleted Tam-R cells. Data are expressed as the mean ± S.D. (n = 3). **(C)** ChIP assays of H3K4me3 and RNA Pol II in TamR cells. Data are mean ± S.D. (n = 3). *P<0.05. **(D)** Chromatin accessibility assay at the *BMP7* locus determined by FAIRE-qPCR analysis in SETD1A-depleted TamR cells. Data are expressed as the mean ± S.D. (n = 4) *P<0.05.

Figure S5

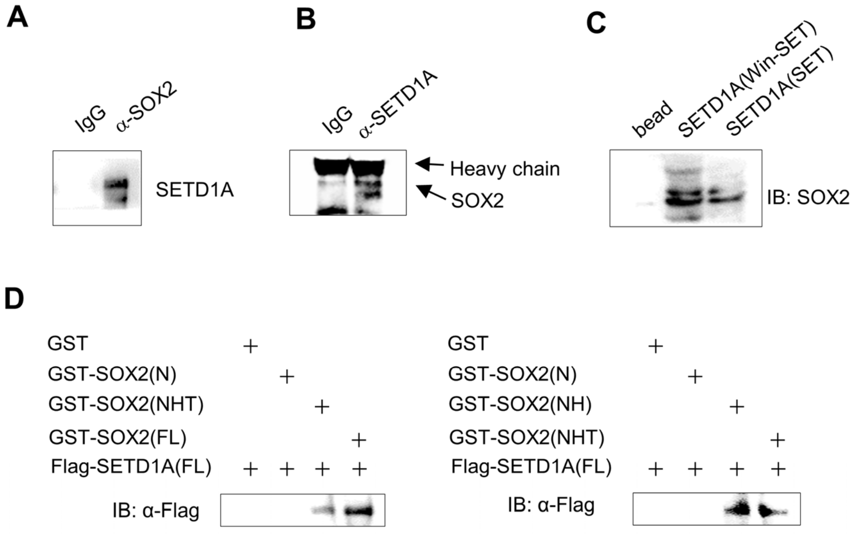

Figure S5. Validation of binding between SETD1A and SOX2. (A, B) The interaction between SOX2 and SETD1A in TamR cells was determined by co-immunoprecipitation as in Figure 6 A&B. **(C)** Direct binding assay using recombinant SET domain of SETD1A. **(D)** Mapping study of SOX2. GST-tagged truncation mutants of SOX2 were expressed in *E. coli* and incubated with FLAG-tagged full-length SETD1A expressed using *in vitro* transcription and translation system. SETD1A bound to the SOX2 fragments was measured using anti-FLAG antibody

Figure S6

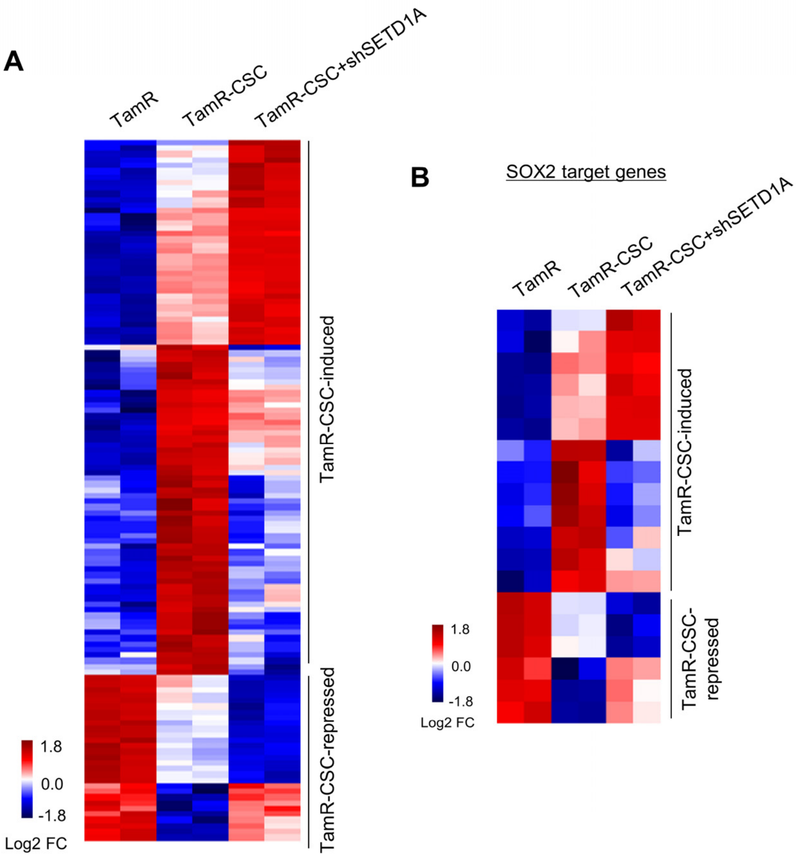

Figure S6. SETD1A regulates CSC-specific gene expression in TamR cells. (A) Heatmap was generated from RNA-seq analysis of TamR and CSC derived from TamR cells expressing either shNS or shSETD1A ($|FC| \geq 1.5$, $P < 0.05$). **(B)** The effect of SETD1A depletion on the expression of SOX2 target gene among CSC-specific genes.

Supplementary Tables

Table S1. Sequences of siRNAs and shRNAs.

Name	Sequence
shNS	CCGGCAACAAGATGAAGAGCACCAACTCGAGTTGGTGCTCTTCATCTTGTTGTTTTT
shSETD1A (1)	CCGGCGGAAGAAGAAGCTCCGATTTCTCGAGAAATCGGAGCTTCTTCTTCCGTTTTTG
shSETD1A (2)	CCGGCTTTGCGGAGAAGAAGCTGTACTCGAGTACAGCTTCTTCTCCGCAAAGTTTTTG
siNS	UUCUCCGAACGUGUCACGUdTdT (sense)<br>ACGUGACACGUUCGGAGAAdTdT (anti-sense)
siSOX2	CAGCUCGCAGACCUACAUGdTdT (sense)<br>CAUGUAGGUCUGCGAGCUG dTdT(anti-sense)

Table S2. Primer sequences for RT-qPCR, FAIRE-qPCR and ChIP.

Name	Forward	Reverse
ITGB5	GGAAGTTCGGAAACAGAGGGT	CTTTCGCCAGCCAATCTTCTC
NFIA	GCAGGCCCGAAAACGAAAATA	TTTGCCAGAAGTCGAGATGCC
PARP12	CCACGGCTCTTGTGCCTTT	TAGTGCCAAACTTGCATTCCC
FOXA2	GGAGCAGCTACTATGCAGAGC	CGTGTTCATGCCGTTCATCC
SOX2	TACAGCATGTCCTACTCGCAG	GAGGAAGAGGTAACCACAGGG
SETD1A	TTTTCCTTCTTGGCCTCTGA	CCAGAAGAGCATGGAGAAGC
MYC	CTCTCAACGACAGCAGCTCG	CAACATCGATTTCTTCCTCATCTTC
BMP7	GGAACGCTTCGACAATGAGAC	GCAGGAAGAGATCCGATTCCC
NANONG	CTCATCAATGCCTGCAGTTTTTCA	CTCCTCAGGGCCCTTGTCAGC
OCT-4	ATGGCATACTGTGGACCTCA	AGCAGCTTGGCAAACTGTTC
KLF4	CCCACATGAAGCGACTTCCC	CAGGTCCAGGAGATCGTTGAA
18S	GAGGATGAGGTGGAACGTGT	TCTTCAGTCGCTCCAGGTCT
GAPDH	TCTGGTAAAGTGGATATTGTTG	GATGGTGATGGGATTTCC
SOX2 enh(−111kb)	GTTCCTGGGCTCAAGCAAT	CCCCGTTCCCAACCTATACA
SOX2 −1kb	TTTGGGTCTCCTAACTTCTA	GTCATTGTTCTCCCGCTCAT
SOX2 0kb	CAGGAGTTGTCAAGGCAGAG	GGAAAATCAGGCGAAGAATA
SOX2 +1kb	CATCACCCACAGCAAATGAC	TTCCTGCAAAGCTCCTACCG
SOX2 −15kb	ACTACTGGTTCCTGATTCCCTCATC	GCAAGTCCGCAAAAGTTGTCTC
MYC −1kb	TAAAATGCCTTTGGGTGAGG	GCCCCACACATGATTTGTTT
MYC TSS	TATTCATAACGCGCTCTCCA	GGGAGGAATGATAGAGGCATAA
MYC +1kb	GGCACTTTGCACTGGAACTT	GGTGCTTACCTGGTTTTCCA
BMP7 −847bp	GAGGAGGAGGTGGGAAGAAC	CCTTCTTACCCCCGCTAGAC
BMP7 +30bp	GCCTCTTGTGCGATCCAG	CCTTCTTCCCGCTCCTCT

Table S3. Antibodies used for western blotting, coimmunoprecipitation, and ChIP assay.

**Antibody**	**Company**	**Catalog number**
SETD1A	Bethyl Laboratories	A300-289A
β-actin	Santa Cruz Biotechnology	sc-47778
Histone H3K4me3	Active Motif	39159
MYC	Santa Cruz Biotechnology	sc-40
Mouse IgG	Santa Cruz Biotechnology	sc-2025
Rabbit IgG	Cell Signaling Technology	2729
Pol II	Merck Millipore	05-623
Flag	Sigma-Aldrich	F7425
SETD1B	Bethyl Laboratories	A302-281A
MLL1	Santa Cruz Biotechnology	sc-20153
SOX2	Thermo Fisher Scientific	MA1-014
NANONG	Active Motif	61419
DyLight 488	Vector Laboratories	DI-1488
DyLight 594	Vector Laboratories	DI-2594
